# 

**Published:** 1872-07

**Authors:** 


Rec'd Dec. 14/73 / June [illegible] O.K.

VOL. 2. JULY 1872. No. 7.

### THE GEORGIA MEDICAL COMPANION.

EDITORS:
T. S. POWELL, M. D., and W. T. GOLDSMITH, M. D.

ASSOCIATE EDITORS:

GEORGIA.
Robert Battey, M D,
R C Word, M D,
W L Kirkpatrick, M D,
James A Long, M D,
W L M Harris, M D,
H L Battle, M D,
W C Musgrove, M D,
L B Bouchelle, M D,
John C Drake, M D,
E F Knott, M D,
Thomas Burdell, M D,
E H W Hunter, M D,
V S Hitt, M D,
J E Pope, M D,
C H Bass, M D,
J A Hunnicutt, M D,
A H Brantley, M D,
J J Knott M D,
J C Wisdom, M D,
W W Leake, M D.

ALABAMA.
J C Knox, M D,
Geo F Taylor, M D,
J B Barnett, M D,
S M Hogan, M D.
D. S Hopping, M.D.
J T Searcy, M.D.
J F Hill, M.D.

MISSISSIPPI.
C B Gallaway, M D,
Edward Lea, M D,
S V D Hill, M D,
W B Harvey, M D,
J W M Shattuck, M D,
E T Henry, M D.
T P Lockwood, M D,
Robert Kells, M D.
R J Preston, M D,

A D Hodge, M D, Ark.

VIRGINIA.
Charles S Lewis, M D,
John H Claiborn, MD,
F Horner, Jr, M D,
A S Payne, M D,
J. E. Lockridge, M. D.
J S Whorton, M D.
Wm O Owen, M D,
Sam'l P. Ripley, M D,
Benj Blackford, M D,
E A Drewry, M D,
Rob. B Tunstall, M D,
A J Terrell, M D,
W. F Barr, M D,
L. B. Anderson, M. D.
S L. Jepson, M.D., W Va
J M Lazzell, M. D.,
Pres't Med. Soc. W. Va.

J K Hodge, M D, Ark.

SOUTH CAROLINA.
E T McSwain, M D.
G M Rivers, M.D.,
J. D. Tucker, M. D., Tenn.
Swan M Burnett, M. D., Tenn.
S M Welch, M D, Tex
T J Heard, M D, Tex.
W A Heard, M D, Tex.

NORTH CAROLINA.
Geo A Foote, M D.
Thos. F Wood, M D.
H Kelly, M. D.
J R Hughes, M. D.
N J Pittman, MD,
S S Satchwell, M D.
A B Pierce, M D,
E A Anderson, M D
H T Bahnson, M. D.
Willis Alston, MD,

CORRESPONDING EDITORS:

J M Toner, M D, Washington City, D C; John H Weir, M D, Ill; Thomas J Gallaher, M D, Pa; Ely Van DeWarker, M D, N Y; Rob't Robson, M.D. Ind; C C F Gay, M D, N Y, (Surgeon of Buffalo General Hospital); W T Taylor, M D, Ky; A Patton, M D, Ind; J Orne Green, Boston, Mass; B W Stone, M D, Ky, (Assistant Physician Western Lunatic Asylum); James Tyson, M D, Philadelphia, Pa; M H Henry, M D, N Y, (Surgeon to New York Dispensary, Department of Venerial and Skin Diseases); Wm R Whitehead, M D, N Y; Thomas H Kearney, M D, Cincinnati, O; Benjamin Howard, M D, New York, Chas Kearns, M D, Ky, S C Busey, M D, Washington, D C.; A W Hobson, M. D., Ark.; A W Calhoun, M. D., now of Berlin, Prussia; T Curtis Smi h, O; George Strawbridge, M D, W W Leen, M D, Philadelphia.

*"QUICQUID PRÆCIPIES ESTO BREVIS."*

ATLANTA, GA.
FRANKLIN STEAM PRINTING HOUSE.
J. J. TOON, PROPRIETOR.

TERMS, - - - - $2 A YEAR, IN ADVANCE

